# Express photolithographic DNA microarray synthesis with optimized chemistry and high-efficiency photolabile groups

**DOI:** 10.1186/s12951-016-0166-0

**Published:** 2016-03-02

**Authors:** Matej Sack, Kathrin Hölz, Ann-Katrin Holik, Nicole Kretschy, Veronika Somoza, Klaus-Peter Stengele, Mark M. Somoza

**Affiliations:** Institute of Inorganic Chemistry, Faculty of Chemistry, University of Vienna, Vienna, Austria; Department of Nutritional and Physiological Chemistry, Faculty of Chemistry, University of Vienna, Vienna, Austria; Christian Doppler Laboratory for Bioactive Aroma Compounds, University of Vienna, Vienna, Austria; Roche Diagnostics, Penzberg, Germany

**Keywords:** Microarray, Phosphoramidite chemistry, NPPOC, Thiophenyl-NPPOC, Photolabile

## Abstract

**Background:**

DNA microarrays are a core element of modern genomics research and medical diagnostics, allowing the simple and simultaneous determination of the relative abundances of hundreds of thousands to millions of genomic DNA or RNA sequences in a sample. Photolithographic in situ synthesis, using light projection from a digitally-controlled array of micromirrors, has been successful at both commercial and laboratory scales. The advantages of this synthesis method are its ability to reliably produce high-quality custom microarrays with a very high spatial density of DNA features using a compact device with few moving parts. The phosphoramidite chemistry used in photolithographic synthesis is similar to that used in conventional solid-phase synthesis of oligonucleotides, but some unique differences require an independent optimization of the synthesis chemistry to achieve fast and low-cost synthesis without compromising microarray quality.

**Results:**

High microarray quality could be maintained while reducing coupling time to a few seconds using DCI activator. Five coupling activators were compared, which resulted in microarray hybridization signals following the order ETT > Activator 42 > DCI ≫ BTT ≫ pyridinium chloride, but only the use of DCI led to both high signal and highly uniform feature intensities. The photodeprotection time was also reduced to a few seconds by replacing the NPPOC photolabile group with the new thiophenyl-NPPOC group. Other chemical parameters, such as oxidation and washing steps were also optimized.

**Conclusions:**

Highly optimized and microarray-specific phosphoramidite chemistry, along with the use of the very photosensitive thiophenyl-NPPOC protecting group allow for the synthesis of high-complexity DNA arrays using coupling times of 15 s and deprotection times of 9 s. The resulting overall cycle time (coupling to coupling) of about 50 s, results in a three-fold reduction in synthesis time.

## Background

DNA microarrays are one of the core technologies for genomic research, allowing scientists access to the full breadth and complexity of genomes in single experiments. Typical microarray experiments focus on quantifying the abundance of nuclear DNA and RNA for insights into gene expression [1] and into the regulation of gene expression via, e.g. epigenetics [2] and micro RNA expression [3]. More recently, DNA microarrays have proved to be valuable beyond hybridization-based assays, for measuring the affinity and specificity of DNA-binding proteins [4, 5], as platforms for aptamer-based multiplexed bioaffinity assays [6–8], and for large-scale oligonucleotide synthesis for assembly into genes [9–11], for targeted sequence capture and enrichment [12, 13], for the rational design of antibody libraries via phage display [14], and for the creation of genome-wide knockout bacterial [15] and cell libraries [16, 17]. The developing technology of RNA microarrays, synthesized directly using phosphoramidite chemistry [18, 19], or synthesized enzymatically from a DNA microarray template [20], and peptide nucleic acids arrays [21] also have important applications in genomics and bioaffinity research, and share many synthesis and technological aspects.

Modern DNA microarrays are synthesized using a variety of in situ methods, all based on modifications of the high-efficiency phosphoramidite chemistry developed by Caruthers and coworkers [22, 23]. The original and still most common approach to in situ microarray synthesis is a derivative of photolithographic technology. The photolithographic method is based on the use of optical imaging systems to deliver light to the synthesis surface, where array layout and sequences are determined by selective removal of the photocleavable protecting groups on the terminus of each oligonucleotide. The primary advantages of the photolithographic approach are the very high surface density of unique DNA features that can be achieved, and the speed and flexibility of the synthesis chemistry. The flexibility of the approach results with the use of an imaging system centered on a digital micromirror device (DMD) in place of photomasks to deliver patterned light to the synthesis surface. This approach, termed Maskless Array Synthesis (MAS), allows virtual masks to control the layout and the oligonucleotide sequences on the array. The speed of the photolithographic approach is due to the ability to use high efficiency photolabile groups that can be removed quickly with light exposure, and the minimal set-up time for new microarray designs. Early DNA photolithographic synthesis used 5′-(α-methyl-2-nitropiperonyl)oxycarbonyl (MeNPOC) [24] and dimethoxybenzoincarbonate (DMBOC) [25] protected phosphoramidites. The relatively low stepwise yield obtained with these groups has limited their use to microarrays of short oligomers [26]. This limitation was overcome with the discovery of the 2-(2-nitrophenyl)propoxycarbonyl (NPPOC) group, which provides the almost quantitative coupling yield and significantly higher photolysis quantum yield necessary for the synthesis of long oligonucleotide microarrays [27–29]. The principle limitation of the NPPOC group is its low absorptivity (ε_365nm/DMSO_ ≈ 260 M^−1^cm^−1^). These shortcomings of NPPOC have been overcome with a recently developed derivative, thiophenyl-2-(2-nitrophenyl)-propoxycarbonyl (SPh-NPPOC), which has both a higher quantum yield for photodeprotection and a much higher absorptivity [30]. The overall photodeprotection efficiency of SPh-NPPOC is 12 times greater than that of NPPOC. This allows such short exposures that the synthesis time is then dominated by the phosphoramidite coupling reaction and washing steps. Here we report on significant optimizations to the microarray synthesis chemistry which, combined with the new photolabile group, allows for very fast and efficient synthesis of high-density DNA microarrays. The optimization experiments presented here include the evaluation of alternative activators and activator concentrations, the determination of the optimal coupling time, the best oxidation strategy, and other chemical synthesis parameters.

## Results

### Activator optimization

The coupling reaction that extends the oligonucleotide chain by one nucleotide unit relies on the nucleophilic substitution, by the terminal 5′-hydroxyl group, of the diisopropylamino group of the phosphoramidite [31]. This reaction requires the involvement of an activator, first to protonate, and then to displace the nitrogen of the leaving group, resulting in a reactive intermediate vulnerable to nucleophilic attack by the nucleosidic alcohol. Thus, the reaction rate should benefit from more acidic and more nucleophilic activators. Many activators have been developed with the aim to increase the speed of the reaction, particularly for sterically hindered phosphoramidites, i.e., RNA phosphoramidites. We have successfully used 4,5-dicyanoimidazole (DCI) [32] as an activator in microarray synthesis for many years, but decided to test alternative activators that might allow for faster coupling. One of the key differences between standard solid-phase synthesis of oligonucleotides and photolithographic synthesis is that solid-phase synthesis relies on the use of the acid-labile dimethoxytrityl (DMT) 5′-hydroxyl protecting group, and is thus somewhat sensitive to very acidic activators, which can prematurely remove some DMT groups, leading to *n*+1 errors [33]. The photolabile groups are not limited in this manner, suggesting that very acidic activators could be useful alternatives if they reduce microarray synthesis time. DCI itself is known as an effective activator even though it has a relatively high pK_a_, presumably because it is a better nucleophile [32]. In order to determine if alternative activators could be used to shorten the coupling time, we tested and compared five activators which have been suggested in the literature as effective activators: DCI, 5-ethylthio-1*H*-tetrazole (ETT) [34], 5-benzylthio-1*H*-tetrazole (BTT) [35], 5-[3,5-Bis(trifluoro-methyl)—phenyl]-1*H*-tetrazole (Activator 42) [36], and pyridinium chloride [37].

**Fig. 1:**
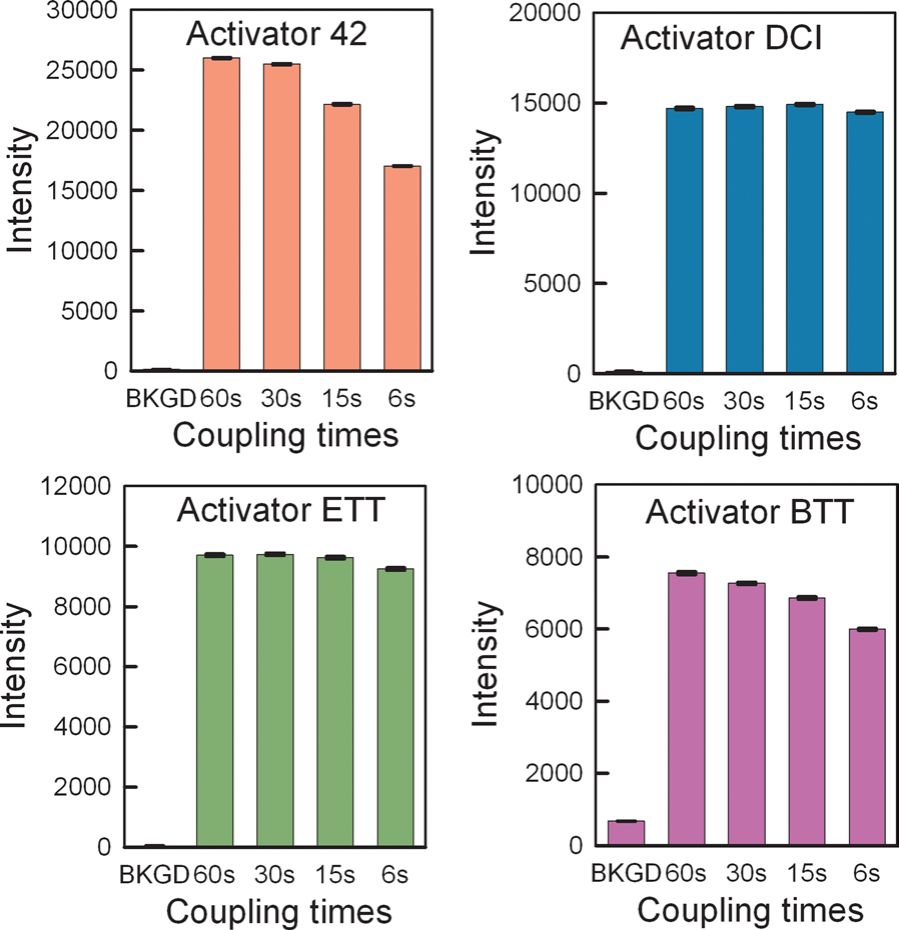
The optimal coupling times for Activator 42, DCI, ETT and BTT were determined in microarray synthesis and hybridization experiments. DCI and ETT activators result in maximum hybridization signal at very short coupling times whereas the hybridization signal from arrays synthesized with BTT and 42 increases with coupling time. *Error bars* are the SEM

**Fig. 2:**
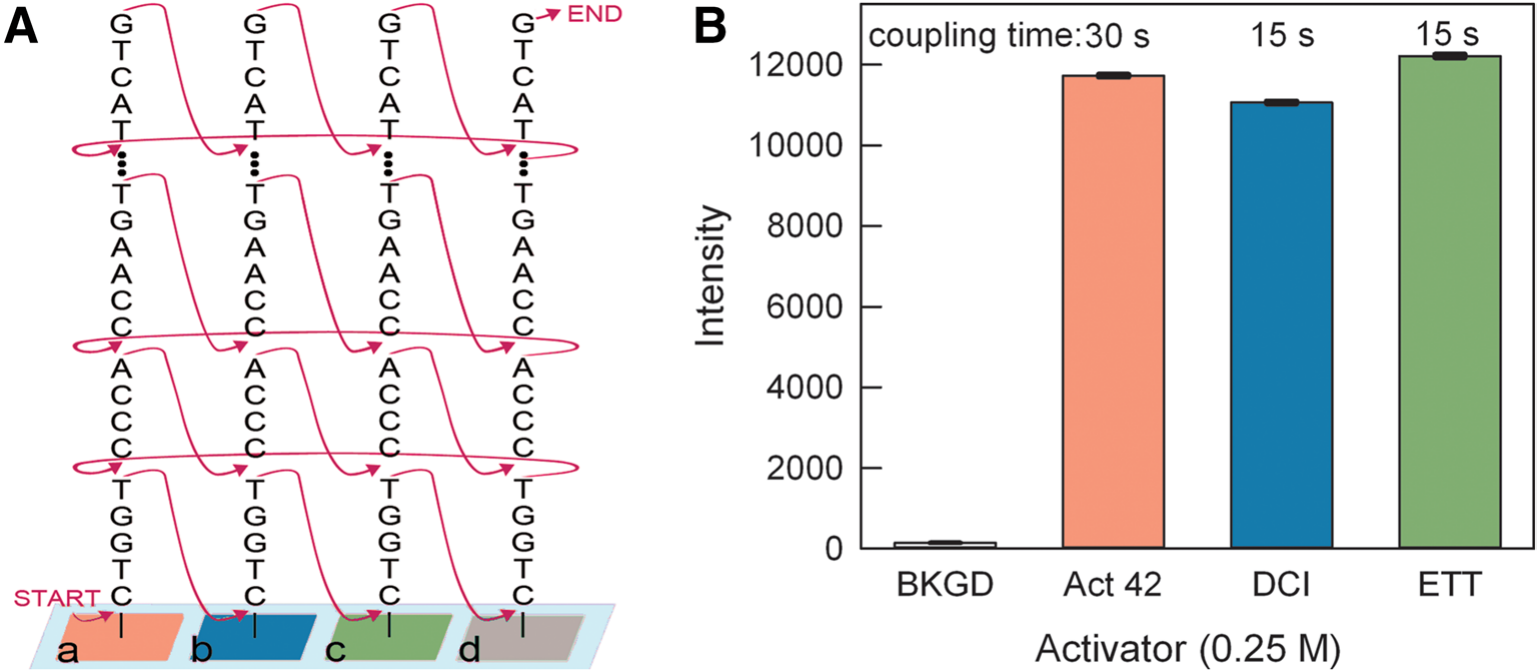
Direct comparison activators by synthesizing sets of mixed base 25-mers on a single microarray surface. Each oligonucleotide set was synthesized using a different activator. To avoid order-of-synthesis effects, all sets were synthesized approximately in parallel using the scheme shown in** A**. The results for Activator 42, DCI and ETT  (*Graph* **B**) were evaluated using the intensity values obtained by hybridizing with a Cy3-labeled complementary oligonucleotide

The first experiments were to determine, for each of the five activators, the optimal coupling time. For these experiments, the same DNA sequence was synthesized four times on the microarray surface, with a different coupling time: 60, 30, 15 and 6 s. The resulting microarrays were hybridized with the fluorescently labeled complementary sequence and scanned. High fluorescence signal was taken as a proxy for effective coupling during synthesis. Microarray synthesis using pyridinium chloride as the activator resulted in very low signal (data not shown). The results for the remaining four activators tested are graphed in Fig. 1. All activators produced microarrays with high hybridization signal/noise, except for BTT, which resulted in arrays with relatively low signal and high background noise. Activator 42 resulted in the highest signal/background, but the signal was strongly dependent on coupling time, with the shorter coupling times resulting in relatively weak hybridization values. Synthesis with both DCI and ETT also resulted in strong hybridization signals, but with the advantage that the strong signal could also be obtained with very short coupling times, under 15 s. Because it is difficult to make sufficiently accurate absolute hybridization intensity comparisons between microarrays, a synthesis was designed to result in a microarray with a single DNA sequence, but with three or four sets of replicates, each replicate set synthesized using a different protocol. The sets were synthesized approximately in parallel, as shown in Fig. 2A, in order to minimize order-of-synthesis effects. When the syntheses are performed in series, the oligonucleotides synthesized first hybridize more weakly, indicating that exposure to synthesis reagents degrades the DNA or the chemical bonds linking the DNA to the glass surface. For multiple 25-mers synthesized in series, the hybridization intensity drops by ~6 % relative to that of the subsequently synthesized oligonucleotide. This observed degradation indicates that minimizing synthesis time also results in higher quality microarrays.

**Fig. 3:**
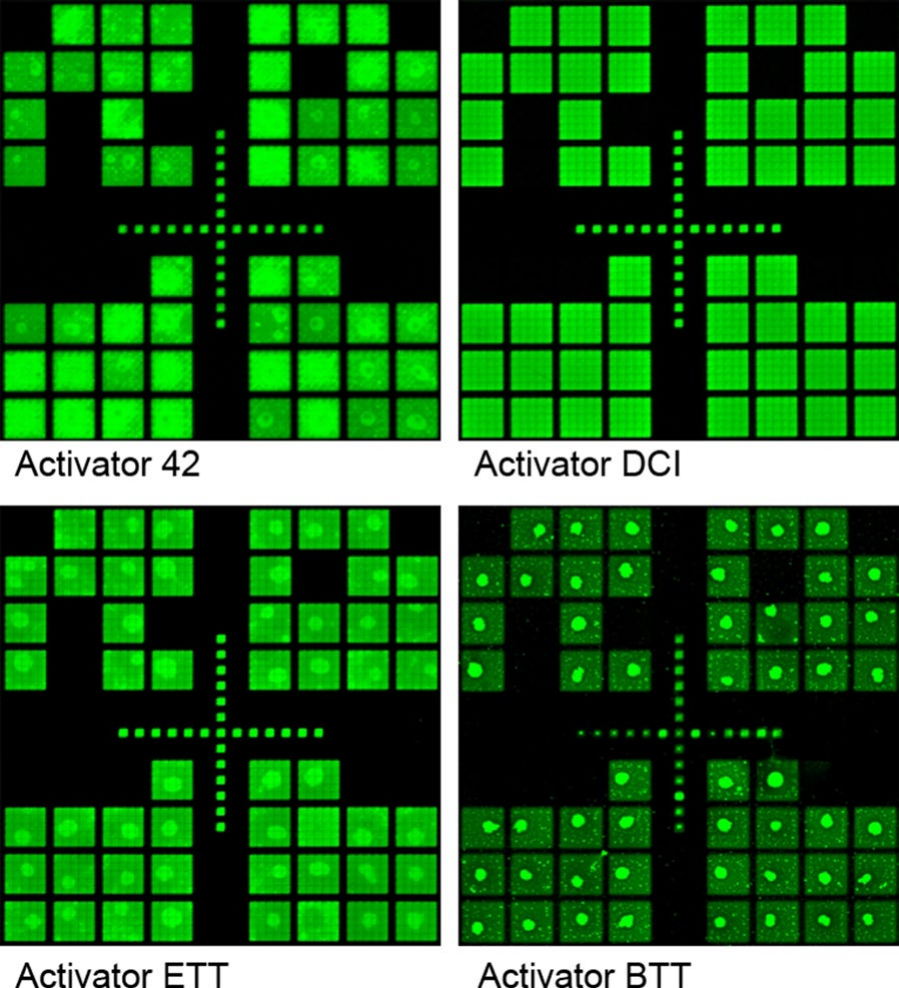
Scan image details of microarrays synthesized using four different activators. Only the use of DCI activator resulted in microarrays with highly homogenous features. The small features are 14 × 14 µm and the large rectangles are made up of an array of 5 × 5 of the smaller features

These coupling results were then used to design an experiment allowing a direct comparison between the three best activators, Activator 42, ETT and DCI. Based on the results shown in Fig. 1, the coupling times were chosen to be 30 s for Activator 42, and 15 s for both DCI and ETT. The hybridization intensity results from this microarray synthesis are shown in Fig. 2B. The results are similar for all three activators but follow the order ETT > Activator 42> DCI, which indicates that ETT is the better choice since it results in a slightly higher hybridization intensity than Activator 42 while requiring only half the coupling time. However, a visual examination of the scan images used to generate Fig. 1 indicated activator-specific effects on the intra- and inter-feature intensity homogeneity. Figure 3 shows a detail of the center of microarrays synthesized using Activator 42, DCI, ETT and BTT. Except for the array synthesized with DCI, all of the arrays have bright spots which appear to have resulted from the final drying step after hybridization. We speculate that the more acidic activators modify the wetting properties of the surface, making it more susceptible to spot formation. The spot formation was observed consistently with multiple arrays synthesized with Activator 42, ETT and BTT. It may also be possible to avoid this issue by using an alternative surface functionalization, or a different hybridization washing and drying protocol, however, it appears from the images in Fig. 3 that at least some of the hybridization intensity measured for Activator 42, ETT and BTT originates in the drying spot rather than from the actual hybridization signal. Based on these results, we decided to retain the use of DCI activator, but to reduce the coupling time from 1 min to 15 s.

**Fig. 4:**
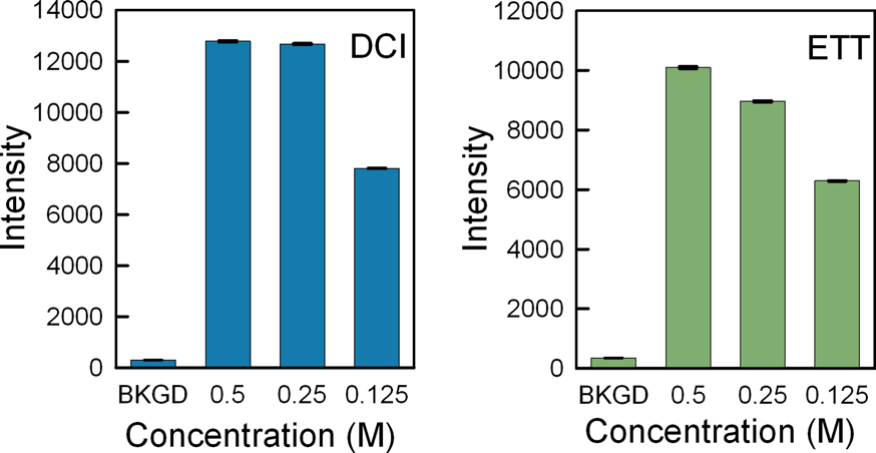
The effect of DCI and ETT activator concentration on hybridization intensity. Higher ETT concentration results in higher hybridization signals, but for DCI, 0.25 M is sufficient to achieve the highest hybridization signal value

It may be possible to reduce the coupling reaction time by increasing the concentration of the activator. Conversely, decreasing the concentration might be beneficial as well, particularly since the 0.25 M standard used in solid-phase DNA synthesis may be too high given the relatively low phosphoramidite concentration (30 mM) we use in microarray synthesis. Low monomer concentrations can be used in microarray synthesis because each microarray includes only about 20 pmol [38] of oligonucleotides, and only about one quarter of these need to be extended with the corresponding phosphoramidite during any given synthesis cycle. This synthesis scale is 3–4 orders of magnitude smaller than the smallest scale normally used in solid phase synthesis. A 30 mM phosphoramidite concentration is amply sufficient at this low scale while providing a margin to protect against incidental water contamination. To determine if the coupling reaction can be improved by increasing or decreasing the activator concentration, we synthesized microarrays using the scheme depicted in Fig. 2a, but using different concentrations of the same activator instead of different activators. DCI and ETT were tested in separate experiments. The results are shown in Fig. 4. The results indicate that a lower activator concentration (0.125 M) does not work as well. A higher concentration (0.5 M) does result in higher hybridization intensity in the case of ETT. For DCI, 0.25 M is close to optimal.

### Oxidation

**Fig. 5:**
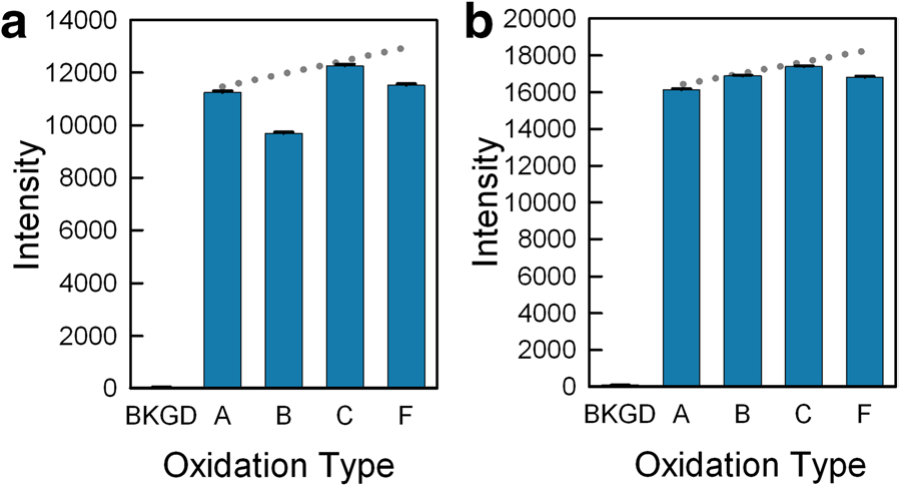
Oxidation optimization. *Graph* **a** shows the hybridization intensity features on a microarray synthesized with a long oxidation step in each cycle (A), a long oxidation step in each cycle but no helium drying step (B), a short oxidation step in each cycle (C), and a single final oxidation (F). Values below the expected trend (*dotted line*) indicate worse results. In *Graph* **b**, *bars* A, B and C are short oxidations steps in each cycle and F is a single final oxidation

Conventional solid phase synthesis of nucleic acids requires an oxidation step preceding the removal of the DMT group because otherwise the acidic deblocking solution would cleave the phosphite triester formed by the coupling reaction. The phosphate triester is stable and rapidly formed by iodine oxidation in the presence of water and pyridine. In photolithographic microarray synthesis, the use of the oxidizer solution can be minimized because the photodeprotection step does not affect the phosphite triester. A final oxidation is still necessary before removing the protecting groups at the end of the synthesis. This minimal oxidation has the advantage of lowering synthesis time and reducing the risk of low coupling yield due to the high water content of the oxidizer. Previously, we had determined that intermittent oxidization or a single final oxidation was slightly preferable to an oxidation in every cycle [39], but decided to revisit this issue. While DCI is not sufficiently acidic to cleave the phosphite triester, ETT, Activator 42 and BTT are [36], therefore requiring oxidation in each cycle. To minimize the oxidation time, as well as water contamination, we compared using an oxidation in the final synthesis cycle only against the same oxidation protocol, but applied in every synthesis cycle. We also tried a very short oxidation exposure in each cycle. Figure 5 shows the results of these experiments. Since the oxidation reagent affects the entire array surface, the scheme shown in Fig. 2a cannot be used to distinguish between oxidation protocols. Instead, four sets of 25mer probes with the same sequence were synthesized in series. As mentioned above, when probes are synthesized in series, rather than in parallel, the probes synthesized earlier are damaged by ongoing reagent exposure and do not hybridize as well as probes synthesized later. The dotted lines in the figure represent this ~6 % trend in increased hybridization intensity of probes synthesized later. The results show that very short oxidations steps, using three pulses (~36 µL) of oxidizer, delivered in one second, are as effective as the oxidations steps 10 times longer (~360 µL in 10 s.). Also, the short oxidation steps in each cycle result in microarrays with higher hybridization signal, as compared with the use of a single, final oxidation. The more frequent oxidation increases the total synthesis time slightly. For a typical gene expression microarray of 60-mers, synthesized with 160 cycles, the total oxidation time would only amount to 160 s, compared to 40 s with the previous method of oxidizing for 10 s after every 40 cycles.

### Drying

**Fig. 6:**
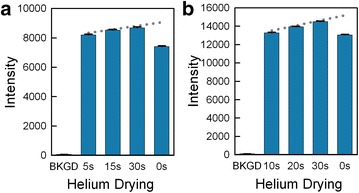
Microarray drying step optimization. Two microarrays synthesized with a variety of helium drying times (5, 15, 30 and 0 s in *Graph* **a**; 10, 20, 30, and 0 s in *Graph* **b**) indicate that the drying step between coupling and light exposure significantly increases hybridization intensity, but that even short drying times are effective

For reasons that remain still unknown, the microarray synthesis benefits from a drying step before the photodeprotection exposure [39]. In this step, an inert gas such as helium is flowed over the glass surface until it is dry. Previously, we have used a 30 s helium drying step, but decided to try to reduce this in order to be able to synthesize microarrays more quickly. Also, since the synthesis is now performed in a reaction chamber with a reduced depth (50 vs. 70 µm), in order to make two arrays simultaneously [40], the drying should be faster. To determine the optimal helium drying time, we synthesized, as with the oxidation tests, four sets of 25mer probes with the same sequence in series on the same array. For one microarray, the drying times were 5, 15, 30 and 0 s (Fig. 6a), and for another array the drying times were 10, 20, 30 and 0 s (Fig. 6b). The dotted line in each graph shows the expected ~6 % trend in increased hybridization intensity of probes synthesized later. The data indicate that, while no drying (0 s) results in a drop of almost 20 % in the hybridization intensity, any drying time above 5 s works equivalently, allowing for a significant reduction of synthesis time. Bar B of Fig. 5a, representing the hybridization intensity of features also synthesized without a drying step shows a similar reduction.

### Photodeprotection of SPh-NPPOC vs. NPPOC

The photodeprotection step of the synthesis has been the most time consuming. The NPPOC photolabile group removal requires a radiant exposure of approximately 6 J/cm^2^, which can be achieved with a 75 s exposure using 80 mW/cm^2^. Significantly higher irradiances are difficult to achieve because, while the total power emitted by arc sources increases with larger lamp size, the larger arc size of larger lamps actually results in less usable light due to the low numerical aperture (NA) of the synthesizer optics. Low NA is necessary to reduce synthesis errors due to scattered light, which scales approximately with NA^2^ [41].

**Fig. 7:**
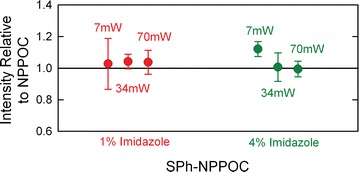
Hybridization intensity values for microarrays of 25-mers synthesized using SPh-NPPOC relative to the equivalent arrays synthesized using NPPOC. Synthesis was carried out using an exposure solvent consisting of either 1 or 4 % imidazole in DMSO, and at radiant power values of 7, 34, or 70 mW/cm^2^

Recent results with SPh-NPPOC have demonstrated that it has a 12-fold greater photolytic efficiency vs. NPPOC [30]. This allows for a faster deprotection or a less intense light source, or a combination of these two parameters. Figure 7 shows that the photolysis of SPh-NPPOC can be carried out successfully over a wide range of radiant power values. Specifically, we used a radiant exposure of 0.5 J/cm^2^ and radiant power values of either 7, 34, or 70 mW/cm^2^, corresponding to exposure times of 70, 15, and 7 s, respectively. We hypothesized that for the shortest exposure times with SPh-NPPOC, the exposure solvent might be more effective with a higher concentration of imidazole. This is because NPPOC— and presumably SPh-NPPOC—photolysis proceeds via a photo-induced β-elimination pathway which requires a small amount of base, preferably an amine base such as imidazole or *N*,*N*-diisopropylethylamine [28, 42]. The proton abstraction rate could be limiting under fast deprotection conditions. The data in Fig. 7 shows that 1 % imidazole is sufficient even for the 7 s photodeprotection.

### Gene expression microarrays

The results from the optimization experiments described above indicate that the microarray synthesis time can be greatly reduced. Specifically, DCI still appears to be the best choice of activator, but the coupling time can be reduced to 15 s. The optimized oxidation protocol, oxidize for ~1 s each cycle, improves the hybridization intensity by about 10 % without significantly extending the synthesis time. The helium drying time can also be reduced to 5 s or perhaps less. Using SPh-NPPOC allows the exposure time to be reduced to about 10 s or less. Since the individual optimizations were tested independently, it was important to determine if they could be successfully combined into a synthesis protocol for making useful microarrays.

**Fig. 8:**
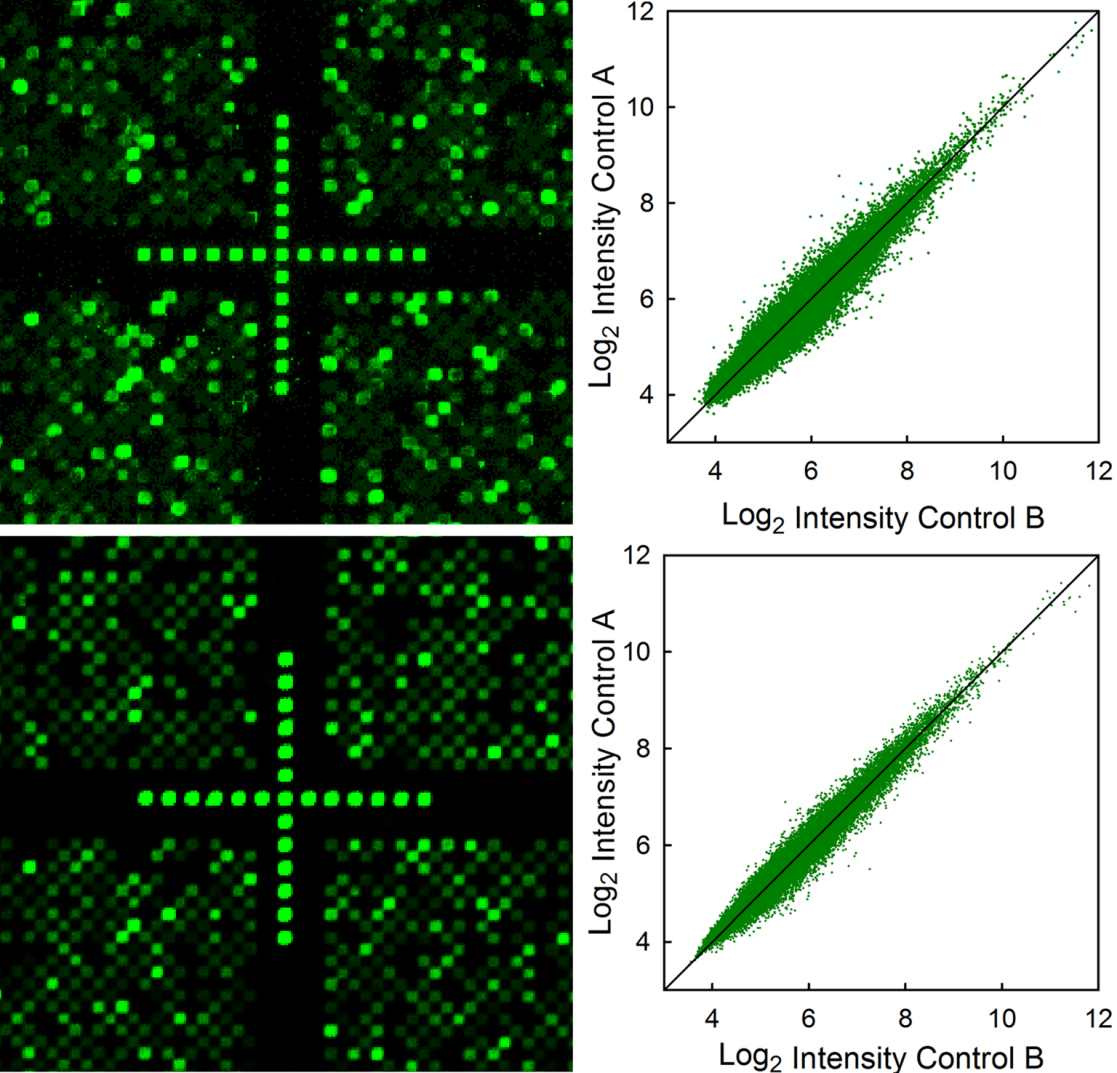
*Left* Details of 2.5 mm resolution scan images from gene expression microarrays synthesized with the Legacy method (*top*) and the Express method (*bottom*) and hybridized with Cy3-labeled cDNA and synthetic spike in controls. The size of each square is ~14 × 14 µm. *Right* Scatterplots of the RMA-processed expression data from the gene expression microarrays synthesized with the legacy method (*top*) and the express method (*bottom*)

Among the most complex and demanding microarray synthesis is that for high-density gene expression microarrays. Using the optimized protocol, we synthesized two sets of gene expression microarrays. One set was made using the legacy protocol with 60 s coupling, 30 s helium drying, NPPOC photodeprotection with 6 J/cm^2^, and an oxidation every 40 cycles and at the end (“Legacy” synthesis). Another set of gene expression microarrays of the same design was made using the new protocol with 15 s coupling, 10 s drying with helium, a short oxidation each cycle, and 10 s SPh-NPPOC photodeprotection (“Express” synthesis). The design of the gene expression microarray included two replicates of each of at least 3 unique 60-mer probes for more than 45,000 human genes. In addition, 20–100 replicates of several quality control and reference sequences were also included. Using a checkerboard-like layout, one-half of the available synthesis features were used to generate a total of 382,536 probe and control oligonucleotides. The microarrays were tested by hybridization with Cy3-labeled cDNA produced from mRNA extracted from a human colon adenocarcinoma cell line (Caco-2). Table 1 summarized the quality control metrics (from Cy3-labeled synthetic spike-in oligonucleotides) from these experiments. Both approaches result in high quality data, but the Express synthesis method is ~3 times faster. Figure 8 shows details of the images, along with the corresponding log_2_ scatter plots of the probe-level data normalized using the robust multiarray average (RMA) procedure [43].

**Table 1:** Quality control data for labeled synthetic spike-in oligonucleotides used for quality control in the hybridization of the gene expression microarrays

	Legacy synthesis		Express synthesis	
	*Average intensity*	*C*_*v*_	*Average intensity*	*c*_*v*_
QC-25mer	1386	0.18	2265	0.20
EcoBioA1	1437	0.27	2118	0.21
EcoBioD2	3254	0.22	2274	0.19
	*Expression*	*SE*	*Expression*	*SE*
QC-25mer	1066	0.97	2263	0.97
EcoBioA1	1102	0.97	2220	0.97
EcoBioD2	2741	0.97	2372	0.97

Average intensity and coefficient of variation (c_v_) for raw intensity data (top), and expression and standard error (SE) values for Robust Multi‐array Average (RMA) normalized data (bottom)

Although the standard error values from the RMA normalized data are the same for both synthesis methods, the image quality for microarrays synthesized using the Express method appear to be consistently better than what we typically achieve using the Legacy method. This is also visible in Fig. 8, where the features from the Express microarray scan have a more homogeneous morphology. This difference may be a result of the shorter synthesis time for the Express synthesis, which reduces the chance that temperature drifts can cause slight changes in the alignment of the optical system. Such drifts can be mitigated by actively realigning the optics during synthesis using an image locking system [44], but such systems significantly increase the complexity of the synthesizer and increase synthesis time.

In order to be able to use the gene expression data to evaluate microarray quality, the two microarrays synthesized with each method were hybridized with cDNA from untreated cells. The deviations from the diagonal line in the scatter plots in Fig. 8 indicate the noise in the expression data rather than differential gene expression. It is clear that the Express method produced microarrays that yield hybridization data with less noise. Although our experience with this new synthesis method is still limited, the faster synthesis consistently generates microarray images with both more consistent spot morphology and reduced noise.

## Discussion

Our objective with this project was to minimize the synthesis time for DNA microarrays without sacrificing quality. For both laboratory scale and industrial scale synthesis, throughput is the main determinant of both cost and productivity. At the same time, the optimization results allow for a better understanding of the underlying phosphoramidite chemistry as well as the photochemistry of the photolabile groups used in the synthesis. Using the synthesis time for human genome-wide gene expression microarrays as a metric, the legacy process we used until a few years ago required 8 to 9 h. Such long syntheses make it impractical to produce more than one or two gene expression microarrays per day per synthesizer. To increase the synthesis efficiency, we first introduced a method to double the efficiency by designing a photochemical reaction cell that could position two glass surfaces at the focus of the optical system [40]. One glass surface serves as the optical entrance to the reaction cell and one serves as the exit. Separated by ~50 µm, the inner surface of each glass slide receives a microarray (mirror images of each other) during the synthesis, and without any change in the synthesis chemistry or synthesis time (the setup time is ~2 min longer). The main limitation of this method is that the two microarrays must share a common design. With the advent of the SPh-NPPOC group, it became apparent that much shorter light exposures were possible, and served as an incentive for optimizing and shortening the remaining chemistry.

**Fig. 9:**
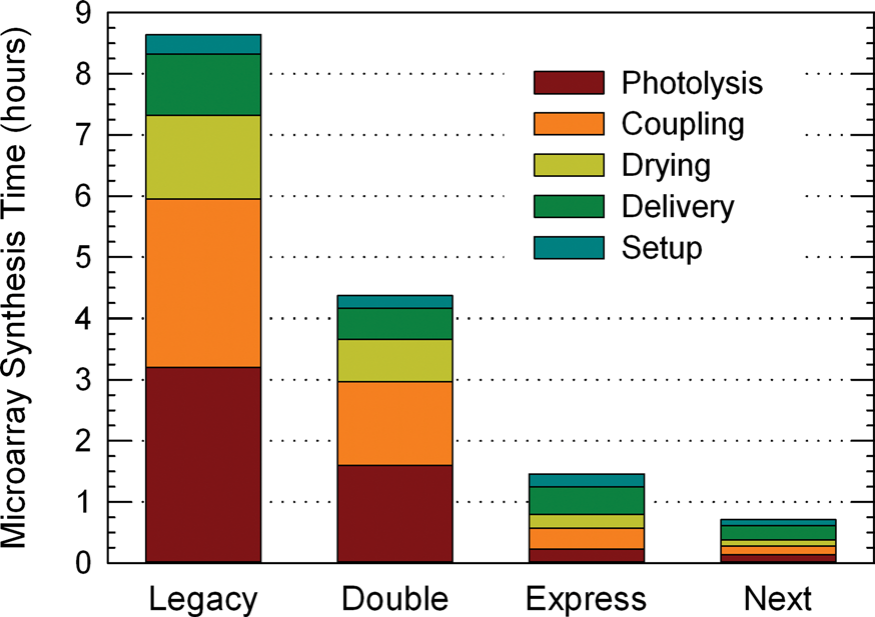
Synthesis time for high-density gene expression microarrays. The optimizations presented here are labeled as “Express”

Figure 9 summarizes the typical synthesis times for gene expression microarrays, and includes the principle steps of each synthesis and their contribution to the overall use of time. Photolysis in the legacy method, along with coupling, dominate the original synthesis time (“Legacy”). The simultaneous synthesis of mirror image arrays (“Double”) reduces the per array synthesis time in half even though the actual synthesis protocol is unchanged. The new optimization efforts presented here is referred to as “Express”. Express synthesis uses a 15 s for coupling, 10 s for drying, and a 10 s for light exposure. With these short times, the previously relatively unimportant contributions to synthesis time, setup time and solvent/reagent delivery times become a significant fraction of the total. The nucleic acid synthesizer we use, an Expedite 8909, has a maximum delivery rate of 1/3–1/4 s per pulse (~12 µL/pulse), so that untimed steps, such as oxidizing, filling the reaction chamber with exposure solvent, or washing with acetonitrile, contribute measurably to the total synthesis time. For the Express synthesis, the volume of some of the washing steps was reduced, but “delivery” is now the largest contributor to the synthesis time. Nevertheless, the new per array synthesis time amounts to about 1.5 h; an almost sixfold reduction compared the legacy method we were using until recently.

Even with these significant reductions in synthesis time, there is still considerable room for further reductions. In the case of coupling time, Fig. 1 indicates that just 6 s is almost as good as the 15 s used for the express synthesis. It appears likely that relatively easy process changes, such as increasing the synthesis temperature by a few degrees, or increasing the phosphoramidite concentration above 30 mM, would allow the coupling reaction to be reduced to just a few seconds. Currently, the reaction is performed at a relatively cool room temperature of 22 °C. Increasing the room temperature, or partially enclosing the reaction chamber and heating it to ~30 °C would likely be sufficient. The results presented in Fig. 6 indicate that the drying step remains effective after another twofold time reduction, from 10 to 5 s. The exposure time can also be further reduced; for the Express synthesis, we used 10 s exposures at a radiant power of 50 mW/cm^2^, but as Fig. 7 shows, higher radiant power could be used to reduce the exposure time to 7 s, or less with a more intense light source. Reducing the delivery and setup times is also possible. These two categories now account for about one-half of the total synthesis time. Some or all of the washing may be unnecessary legacies of the original protocols, developed for solid phase synthesis, where the large surface areas of controlled pore glass require greater washing volumes. For example, it is known that the pyridine in the oxidation solution is a powerful quencher of the detritylation reaction [45], but in photolithographic synthesis, traces of oxidation solution probably will not interfere with photodeprotection, and therefore washing may be unnecessary between these two steps. The setup time could also be reduced by using a second reaction chamber that could be assembled and tested while another synthesis is running. The time savings from these hypothetical reductions have been estimated and are shown in Fig. 9 as “Next”, with an average synthesis time for gene expression microarrays of about 45 min.

Beyond “Next”, it may be challenging to make further large improvements without new development in the chemistry, such as even more efficient photolabile groups or better activators. Nevertheless, a further factor of ~2 can be achieved with a straightforward engineering solution. Three microarrays, of the same or different designs, can be synthesized on the same 25 × 75 mm slide surface by translating the reaction cell assembly and making three consecutive exposures. Since the exposure time needed by the SPh-NPPOC phosphoramidites is only a few seconds, and the remaining steps would be carried out simultaneously for all three arrays, the synthesis time would only be about 50 % longer than synthesis with a single exposure. The solvent and reagent consumption would also increase about 50 % due to the increased size of the reaction chamber. The resulting microarrays could then be independently hybridized.

While increasing the speed of synthesis was the primary goal of this project, it appears that microarray quality improves with the shorter synthesis time. Several of the experiments, such as those depicted in Figs. 5, 6, clearly indicated that one or more of the chemicals used in the synthesis partially degrades the oligonucleotides on the surface. This suggested that faster synthesis would result in improved microarray quality. This does appear to be the case. Although the quality assessment metrics we used to evaluate the gene expression microarrays (Table 1) indicate that the two synthesis methods yield similar microarrays, other metrics, such as spot morphology and the scatter in the gene expression data indicate that the Express synthesis produces higher quality microarrays.

## Conclusions

Optimizing microarray-specific phosphoramidite chemistry and using phosphoramidites with the highly efficient thiophenyl-NPPOC (SPh-NPPOC) photolabile group results in a large reduction in synthesis time without any loss of microarray quality. Combined with previous optimizations, the optimized method allows for high-density arrays of 60-mers to be synthesized in about 90 min. The results also indicate that significant further improvements should be able to reduce synthesis time to less than 30 min per independently hybridizable microarray of 60-mers.

## Methods

### Substrate preparation

All microarrays were synthesized as mirror image pairs using a method published earlier. [40] Briefly, half of the Schott Nexterion Glass D microscope slides (75 × 25 × 1 mm) require two holes with a diameter of approximately 1 mm that serve as the entrance and exit to the reaction chamber defined by one drilled slide and one undrilled slide separated using a 50 μm gasket (Gasoila Industrial Strength SD PTFE tape). The gasket is cut from the tape using a laser cutter. The glass slides are cleaned in an ultrasonic bath prior to functionalization.

### Functionalization of substrates

The glass slides were functionalized with *N*-(3-triethoxysilylpropyl)-4-hydroxybutyramide [26] (Gelest SIT8189.5). The slides were placed in stainless steel rack and gently agitated in a solution of 2 % (v/v) of the silane and 0.1 % acetic acid in 95:5 ethanol/water. After the 4 h silanization at room temperature, the slides were rinsed twice for 20 min in the 95:5 ethanol:deionized water and cured overnight at 120 °C under vacuum. After cooling to room temperature under vacuum, the slides are stored in a desiccator until use.

### Microarray synthesis

The Maskless Array Synthesizer (MAS) instrument consists of two major systems, an optical system and a chemical delivery system. The chemical side consists of an Expedite 8909 nucleic acid synthesizer, which delivers solvents and reagents to the reaction chamber where the microarray synthesis takes place. The optical system is similar to that of a photolithographic system, but it uses an array of 1024 × 768 digitally controlled mirrors (Texas Instruments 0.7 XGA DMD) in place of photomasks to pattern the ultraviolet light from a mercury lamp.

Light from a 350 W DC ultra-high pressure short arc mercury lamp (Newport 6286) is filtered by two consecutive 350–450 nm dichroic mirrors (Newport 66218). The resulting UV light, consisting of three unfiltered mercury lines (365, 405 and 436 nm), are spatially smoothed in a reflective homogenizing light pipe, and imaged onto the DMD. Light reflected by the DMD mirrors are imaged onto the two glass substrates using Offner relay optics. The intensity of UV light reaching the reaction cell image is adjusted according to the readings from a calibrated SÜSS intensity meter with a 365 nm probe (SÜSS MicroTec 1000).

The pattern of mirrors displayed on the DMD are imaged onto the two synthesis surfaces, where they determine microarray layout and oligonucleotide sequences by selectively removing the photolabile protecting groups, either NPPOC or SPh-NPPOC. Reagent delivery and the light exposures are controlled and synchronized by a computer. The phosphoramidite chemistry is similar to that used for solid-phase synthesis. The primary difference is the use of phosphoramidites with a 5‘-OH photolabile protecting group. Upon absorption of a photon near UV photon, and in the presence of a weak amine base (typically 1 % imidazole in DMSO), the NPPOC or SPh-NPPOC group comes off, leaving the 5′ terminal hydroxyl, which reacts with activated phosphoramidite during the next coupling cycle. Representative synthesis protocols are shown in Table 2 for the legacy synthesis and in Table 3 for the express synthesis.

**Table 2:** Representative chemical synthesis protocol, in Expedite 8909 format, used for the legacy syntheses

Cycle NPPOC-dT (legacy synthesis)				
Function	Mode	Pulses	Sec	Description
$Coupling				
1/*Wsh	Pulse	20	0	Flush with Wsh
2/*Act	Pulse	6	0	Act
21/*T + Act	Pulse	5	0	T + Act
2/*Act	Pulse	6	0	Push with Act
1/*Wsh	Pulse	3	60	Couple monomer
1/*Wsh	Pulse	10	0	Flush with Wsh
$Capping				
40/*Gas A	Pulse	1	30	Dry column
$Oxidizing				
15/*Ox	Pulse	30	0	Ox to column
12/*Wsh A	Pulse	45	0	Flush with Wsh A
17/*Aux	Pulse	15	0	Exposure solvent
130/*Event 2 Out	Na	4	1	Event 2 out
17/*Aux	Pulse	10	50	Exposure solvent
12/*Wsh A	Pulse	5	20	Flush with Wsh A
130/*Event 2	Na	4	1	Event 2 out
12/*Wsh A	Pulse	20	0	Flush with Wsh A

The 5′-NPPOC light-deprotection step takes place between the two “Event Out” commands, which trigger the opening and closing of a shutter blocking the UV light. The 70 s exposure, at 80 mW/cm^2^, corresponds to an exposure of 5.6 J/cm^2^. The oxidation, the first two steps after the $Oxidizing header, are used intermittently (every ~40 cycles) and after the last coupling

**Table 3:** Representative chemical synthesis protocol, in Expedite 8909 format, used for the express syntheses

Cycle SPh-NPPOC-dT (express synthesis)				
Function	Mode	Pulses	Sec	Description
$Coupling				
1/*Wsh	Pulse	10	0	Flush with Wsh
2/*Act	Pulse	6	0	Act
21/*T + Act	Pulse	5	0	T + Act
2/*Act	Pulse	6	0	Push with Act
1/*Wsh	Pulse	3	15	Couple monomer
1/*Wsh	Pulse	10	0	Flush with Wsh
$Capping				
40/*Gas A	Pulse	1	10	Dry column
$Oxidizing				
15/*Ox	Pulse	3	0	Ox to column
12/*Wsh A	Pulse	10	0	Flush with Wsh A
17/*Aux	Pulse	15	0	Exposure solvent
130/*Event 2	Na	4	1	Event 2 out
12/*Wsh A	Pulse	5	9	Push with Wsh A
130/*Event 2	Na	4	1	Event 2 out
12/*Wsh A	Pulse	10	0	Flush with Wsh A

The 9 s exposure, at 50 mW/cm^2^, corresponds to an exposure of 0.45 J/cm^2^. The five pulses of Wsh A during the exposure push the exposure solvent towards the waste, but the reaction chamber remains full of exposure solvent until the next washing step. The short oxidation step in this synthesis, the first two steps after the $Oxidizing header, are used after every coupling

After synthesis, the groups are removed by immersing the microarrays in 1:1 (v/v) ethylenediamine and ethanol for 2 h at room temperature, then washed twice in beakers filled with deionized water and dried with argon. Deprotected microarrays were stored in a desiccator cabinet until hybridized.

### NPPOC and SPh-NPPOC Phosphoramidites

NPPOC DNA phosphoramidites were purchased from Sigma-Aldrich (A112N01-01, C114N01-01, G114N01-01, T111N01-01) and diluted to 30 mM with Amidite Diluent (<30 ppm water) from Sigma-Aldrich (L010010-06). SPh-NPPOC phosphoramidites were manufactured by NimbleGen Systems GmbH (Waldkraiburg, Germany) and diluted as above.

### Coupling time optimization

The coupling experiments were performed using the legacy protocol (Table 2), but with different coupling times, 5, 15, 30 and 60 s. In addition, an oxidation step, as indicated in the table, was included in every cycle due to the use of acidic activators. Each microarray was synthesized as shown in Fig. 2a, with each of the replicate sets synthesized using a different coupling time (Fig. 1). The same microarray design and synthesis protocol was used for each activator. Five activators were chosen, DCI in acetonitrile (Biosolve Chimie), 0.25 M ETT (Sigma L0302511), 0.25 M BTT (empBiotech NC-0102 L0302511), 0.25 M pyridine hydrochloride (Fluka 82800), 0.25 M Activator 42 (Aldrich L8300212). Each activator was used at a concentration of 0.25 M in anhydrous acetonitrile when not specified otherwise. After synthesis, the microarrays were deprotected and hybridized as described.

### Activator optimization

The activator optimizations were performed using either 0.25 M DCI, 0.25 M ETT, 0.25 M BTT, 0.25 M pyridine hydrochloride, 0.25 M Activator 42. For the experiments using 0.125 M activator, the activator solution was diluted in anhydrous acetonitrile. For the experiments using 0.5 M activator, the solution was either concentrated by evaporating acetonitrile in a vacuum or (for DCI) by adding the crystalline form (Aldrich 554030).

Nucleic acid synthesizers have separate activator and phosphoramidite ports and mix the activator when needed. The Expedite 8909 accomplishes this mixing by drawing single pulses (~12 µL/pulse) of activator and phosphoramidite in an alternating fashion until reaching the desired volume, 5 pulses of each. This pulse train then mixes in the fluidics system and on the way to the synthesis area. In order to synthesize single arrays using multiple activators (Figs. 3, 4), the normal activator port was used for one of the activators and the other activators or activator concentrations were placed in unused phosphoramidite ports (the Expedite 8909 has nine phosphoramidite ports). In order to mix the activators in these ports with the appropriate phosphoramidite, the normal mixing command in the protocol file (e.g. “21/*T + Act, PULSE, 5, 0”; monomer T and Act simultaneously) was replaced with 10 alternating single port commands, e.g. “11/*T, PULSE, 1, 0”; Monomer T, “7/*T, PULSE, 1, 0”; Monomer 9”. Command seven delivers a single pulse out of port nine, which is filled with another activator. Several control experiments confirmed that this alternative command set resulted in equal coupling efficiency. The effectiveness of the activators was evaluated based on the homogeneity and intensity of the microarray features after hybridization.

### Oxidizer and drying optimization

Since both the oxidation and drying steps affect the entire microarray surface, it was not possible to use the parallel synthesis scheme illustrated in in Fig. 2b. Instead, replicates of the same 25mer sequence were synthesized four times in series. The location of each of several hundred synthesis replicates were randomized across the microarray surface. We observed that, when the same synthesis protocol was used for each of the syntheses, the hybridization intensity increased linearly from the first probe synthesized to the last, with an increase of ~6 % per synthesis. Oxidation or drying protocols resulting in a drop in hybridization signal relative to this trend were judged as inferior. In the case of oxidation (Fig. 5), we evaluated three protocols, a single final oxidation, 10 s oxidation per cycle (30 pulses) and 1 s oxidation per pulse (3 pulses). In all cases, the oxidation solution was tetrahyrofuran/water/pyridine/iodine 90.54/9.05/0.41/0.43 (v/v/v/w) (Sigma-Aldrich L860021). The same analysis was applied to the optimization of the helium drying time. In this case, we tried 0, 5, 10, 15, and 30 s of drying time after the coupling steps.

### NPPOC vs. SPh-NPPOC comparison

In order to make direct comparisons between syntheses using NPPOC vs. SPh-NPPOC, we designed a microarray with probe replicates synthesized using NPPOC phosphoramidites as well as probe replicates synthesized using SPh-NPPOC phosphoramidites. Because all the monomer ports of the Expedite 8909 may not work equally well, the Sph-NPPOC synthesis was performed first, and paused while the monomer ports were cleaned out and filled with NPPOC phosphoramidites. The synthesis of the second set of microarray probes was then continued on the same surface. The location of each set of probes was randomized across the microarray surface. The experiment was performed using 7, 34 and 70 mW/cm^2^ light exposures for the SPh-NPPOC amidites and using 1 or 4 % (w/v) imidazole in DMSO (Fig. 7). Because the probe set synthesized with SPh-NPPOC was performed first, the actual relative performance of SPh-NPPOC is likely ~6 % better than indicated in Fig. 7.

### Genomic cDNA

The human colon adenocarcinoma cell line Caco-2 was obtained from the American Type Culture Collection. Caco-2 cells were cultivated in Dulbecco’s modified Eagle medium supplemented with 10 % fetal bovine serum, 1 % penicillin/streptomycin and 4 mM l-glutamine in a humidified incubator at 37 °C and 5 % CO_2_. After reaching 80–90 % confluence, the cells were seeded into 6-well plates at a density of 4 × 10^5^ cells per well and differentiated into enterocyte like cells for 21 days. During this period the medium was exchanged every second to third day. On day 21, the cells were starved using serum-free Dulbecco’s modified Eagle medium supplemented with 1 % penicillin/streptomycin and 4 mM l-glutamine. After 90 min, the cells were washed with cold PBS prior to RNA isolation following the manufacturer’s instructions (RNeasy Mini Kit, Qiagen). The A_260/280_ and A_260/230_ ratios of the isolated RNA were measured photometrically (Tecan) as quality measures in addition to agarose gel electrophoresis. The RNA (10 µg) was labeled using Cy3-conjugated random nonamer primers (Tebu Bio) in the reverse transcription as described previously by Ouellet et al. [46].

### Microarray hybridization

The microarrays used for the optimization experiments were hybridized in a solution containing 150 µl 2× MES, 110 µl nuclease free water, 13.3 µl acetylated BSA and 26.7 µl of 100 nM 5′-Cy3-labeled complementary sequence. In the case of the microarrays used for the gene expression experiment, the hybridization solution contained 3 µL herring sperm DNA (10 mg/ml), 15 µL acetylated BSA (10 mg/ml), 135 µL 2× MES hybridization buffer, Cy3-labeled cDNA in 85 µL water, 10 µL Cy3-labeled QC 25mer oligo (100 nM), 10 µL Cy3-labeled ECO1BioA1 (100 nM), and 10 µL Cy3-labeled ECO1BioD2 (100 nM). All hybridizations took place in self-adhesive chambers (Grace Biolabs SA200). The microarrays were rotated (~ 30 Hz) in a hybridization oven at 42 °C. An air bubble filling a quarter to a third of the chamber volume moves around the hybridization chamber due to the rotation, circulating and mixing the hybridization solution, which promotes efficient hybridization [47]. After 4 h for the optimization experiments or 22 h for the gene expression experiments, the chamber was removed while submerged in a Petri dish filled with non-stringent wash buffer pre-warmed to 42 °C. The microarrays were washed with vigorous shaking in 50 ml centrifuge tubes filled with 30 ml non-stringent wash buffer (SSPE; 0.9 M NaCl, 0.06 M phosphate, 6 mM EDTA, 0.01 % Tween20) for 2 min., and then similarly washed with stringent wash buffer (100 mM MES, 0.1 M Na^+^, 0.01 % Tween20) for 1 min. Finally, the microarrays were rinsed for about 5 s in final wash buffer (0.1× saline-sodium citrate buffer) to remove most of the salt before being dried using a microarray centrifuge. Microarrays were scanned at 2.5 µm resolution. Feature- or probe-level data was extracted with NimbleScan 2.1 (Roche-NimbleGen).

### Gene expression analysis

The extracted probe-level data was normalized using the robust multichip analysis (RMA) function of NimbleScan 2.1 (Roche-NimbleGen). The normalized intensities were log_2_ transformed and a scatter plot of the two biological replicates created with SigmaPlot 11.0. [48, 49].

### Gene expression microarray quality control

Three synthetic HPLC purified 5′-Cy3-labeled DNA oligomers, were added to the hybridization buffer at concentrations of 3.7 nM. The names and sequences of these oligonucleotides are:

GAC CAG GGT GGT TCA TGA TGA TGA C, QC_25mer.

GAT TTA GGT TTA CAA GTC TAC ACC GAA TTA ACA ACA AAA AAC ACG TTT TGG AG, ECOBioA1t_53mer.

GAA ATG AGG GTG TAA TTG ATT GGG CAA CTG TGC GCC ACG CTA CTT TCT TCT TCG CTT AAC, ECOBioD2_60mer.

The microarray layout was designed with 100 probes for QC_25mer, 140 probes for ECOBioA1t_53mer, and 140 probes for EcoBioD2_60mer. The location of each feature on the microarray was randomized along with all the other probes. Several assessment metrics, based on these synthetic oligonucleotides, were used to evaluate the synthesis and hybridization quality the microarrays. The outcomes of the assessments are summarized in Table 1.

## Abbreviations

BSA: bovine serum albumin
BTT: 5-benzylthio-1*H*-tetrazole
DCI: 4,5-dicyanoimidazole
DMD: digital micromirror device
DMT: dimethoxytrityl
ETT: 5-ethylthio-1*H*-tetrazole
NPPOC: 2-(2-nitrophenyl)-propyloxycarbonyl
MAS: maskless array synthesis
MES: 2-(*N*-morpholino)ethanesulfonic acid
SPh-NPPOC: thiophenyl-2-(2-nitrophenyl)-propoxycarbonyl
SSC: saline-sodium citrate
